# Immunomodulatory and Immunonutritional Effects of a Standardized Extract of Cultured *Lentinula edodes* Mycelia in Cancer: From Prevention to Perioperative Microenvironment Stabilization

**DOI:** 10.3390/nu18162731

**Published:** 2026-08-21

**Authors:** Richi Nakatake, Tetsuya Okuyama, Shigeki Adachi, Toru Matsu-ura, Hiroaki Kitade, Mikio Nishizawa

**Affiliations:** 1 Department of Hepato-Biliary-Pancreatic Surgery, Kansai Medical University Medical Center, 10-15 Fumizono-cho, Moriguchi 570-8507, Osaka, Japan; kitade.hir@kmu.ac.jp; 2 Department of Pancreatobiliary Surgery, Kansai Medical University, 2-5-1 Shin-machi, Hirakata 573-1010, Osaka, Japan; okuyama.tet@kmu.ac.jp; 3 Department of Pathology, Kansai Medical University, 2-5-1 Shin-machi, Hirakata 573-1010, Osaka, Japan; adachi.shi@kmu.ac.jp (S.A.); matsuura.tor@kmu.ac.jp (T.M.); 4 Faculty of Life Sciences, Ritsumeikan University, Kusatsu 525-8577, Shiga, Japan; nishizaw@sk.ritsumei.ac.jp

**Keywords:** AHCC^®^, a standardized extract of cultured *Lentinula edodes* mycelia, ECLM, ischemia–reperfusion injury, natural killer cells, hepatocellular carcinoma

## Abstract

A standardized extract of cultured *Lentinula edodes* mycelia (ECLM), commercially known as AHCC^®^, shows anti-inflammatory, immunomodulatory, and organ-protective properties. Experimental studies have indicated that ECLM modulates innate and adaptive immunity, including natural killer (NK) cell activity, antigen-presenting cell function, T-cell responses, and cytokine balance. These effects are particularly relevant in oncology because surgical stress and ischemia–reperfusion injury (IRI) generate a transient perioperative environment characterized by immune suppression, inflammation, and conditions favorable for metastatic progression. Recent animal studies have demonstrated the protective effects of ECLM in intestinal and hepatic IRI models, providing a potential mechanistic rationale for improving the perioperative host microenvironment. Clinical studies on hepatocellular carcinoma, pancreatic cancer, and gynecological malignancies suggest that ECLM may offer potential benefits in immune preservation, nutritional support, symptom management, and recurrence prevention, although most studies are small and hypothesis-generating. Emerging evidence from patient-derived xenograft and spontaneous carcinogenesis models further suggests that ECLM may influence tumor biology beyond host immune activation, although the underlying mechanisms require elucidation. This review summarizes the mechanistic, preclinical, translational, and clinical evidence supporting the use of ECLM as a candidate for perioperative immunonutritional strategies. We propose the hypothesis that ECLM may function as a perioperative microenvironmental stabilizer that integrates immune preservation and intestinal barrier protection.

## 1. Introduction

Cancer recurrence after apparently curative surgery is a major cause of cancer-related mortality. Although recurrence has traditionally been attributed to tumor-intrinsic characteristics, such as genomic instability, invasive potential, stemness, and therapeutic resistance, accumulating evidence suggests that host-related factors also play a critical role in determining metastatic outgrowth. The immune system continuously eliminates disseminated tumor cells through both innate and adaptive immune surveillance mechanisms, particularly those mediated by natural killer (NK) cells and cytotoxic T lymphocytes [1].

The perioperative period represents a biologically vulnerable interval during cancer treatment. Surgical manipulation may release tumor cells into circulation, while tissue injury, anesthetic exposure, neuroendocrine stress, systemic inflammation, and ischemia–reperfusion injury (IRI) can transiently impair antitumor immunity [2,3,4,5,6]. Consequently, the perioperative phase has emerged as a critical window during which host responses may influence long-term oncological outcomes. This concept has led to the hypothesis that postoperative recurrence is not solely a delayed manifestation of pre-existing micrometastases but may also be shaped by modifiable perioperative biological events [2,3].

A standardized extract of cultured *Lentinula edodes* mycelia (ECLM), commercially known as AHCC^®^ (Amino Up Co. Ltd., Sapporo, Japan), is the extract derived from cultured shiitake mushroom mycelia. Unlike conventional pharmacological immunomodulators, ECLM is administered orally as a nutritional supplement and exerts its biological effects primarily through the gastrointestinal tract. One proposed bioactive fraction consists of low-molecular-weight α-1,4-glucans generated during cultivation, which are thought to have favorable bioavailability and immunological activity [7].

Emerging evidence indicates that ECLM functions not only as an immune modulator but also as an immunonutritional intervention. Clinical studies have reported preservation of immune competence, reductions in chemotherapy-related taste disorders, and maintenance of nutritional parameters in patients receiving ECLM during cancer treatment [8,9,10,11]. Furthermore, experimental studies have demonstrated its protective effects against intestinal and hepatic IRI, indicating its roles in preserving epithelial barrier integrity, attenuating systemic inflammation, and maintaining metabolic homeostasis [12,13]. These findings are particularly relevant in perioperative oncology, where immune dysfunction, intestinal barrier disruption, and organ injury collectively contribute to postoperative complications and cancer recurrence.

Recent advances in translational cancer research have expanded the potential significance of ECLM. Evidence from patient-derived xenograft (PDX) models and spontaneous carcinogenesis studies suggests that ECLM may exert biological effects extending beyond host immune activation alone. These observations support the possibility that ECLM influences both host-related and tumor-intrinsic pathways involved in cancer progression.

Taken together, these independent observations suggest the possibility of a broader conceptual framework in which ECLM acts as a perioperative microenvironment stabilizer, integrating immune preservation, organ protection, intestinal barrier maintenance, and potential regulation of tumor biology. This narrative review summarizes the mechanistic, preclinical, clinical, and translational evidence for the use of ECLM in cancer, with particular emphasis on immunonutrition, perioperative oncology, IRI, and bench-to-bedside development toward precision cancer care. We further explore how ECLM may influence host resilience and tumor-related pathways through interconnected immunological and metabolic mechanisms. In addition to its immunomodulatory activity, ECLM has been reported to exhibit anti-inflammatory properties in several experimental models. Attenuation of excessive inflammatory responses may contribute to the preservation of tissue integrity and the maintenance of a favorable perioperative microenvironment.

## 2. Literature Search Strategy

This narrative review was based on a structured literature search of PubMed, Scopus, and Web of Science. Publications available up to June 2026 were considered. The following keywords and their combinations were used: “AHCC”, “active hexose correlated compound”, “standardized extract of cultured Lentinula edodes mycelia”, “ECLM”, “cancer”, “immunonutrition”, “natural killer cells”, “ischemia–reperfusion injury”, “perioperative”, “hepatocellular carcinoma”, “pancreatic cancer”, and “patient-derived xenograft”.

Original articles, preclinical studies, clinical studies, and relevant review articles published in English were considered. Studies were selected based on their relevance to the immunomodulatory, immunonutritional, organ-protective, and perioperative effects of ECLM. Reference lists of relevant publications were also manually screened to identify additional eligible studies. Owing to the narrative nature of this review, a formal systematic review process or meta-analysis was not performed. As the available literature is limited and heterogeneous, particularly for clinical studies, emphasis was placed on representative mechanistic, translational, and clinical evidence rather than quantitative evidence synthesis.

## 3. Perioperative Immune Suppression and the Metastatic Window

### 3.1. Surgical Stress as a Biological Driver of Metastasis

Major surgery activates the sympathetic nervous system and hypothalamic–pituitary–adrenal axis, leading to catecholamine and glucocorticoid release. These mediators can suppress NK cell cytotoxicity, alter T-cell function, and modify cytokine networks [2,5,6,14]. In experimental models, stress and surgical interventions have been shown to promote tumor development, in part through suppression of NK cell activity [5,14].

Therefore, the perioperative metastatic window is characterized by simultaneous tumor cell dissemination and reduced immune clearance. Surgery may also alter endothelial adhesiveness, coagulation, platelet–tumor cell interactions, and inflammatory signaling, all of which can facilitate tumor cell survival and implantation [2,3]. This biological framework suggests the development of perioperative interventions that preserve host immune surveillance rather than focusing solely on tumor-directed cytotoxic therapy.

### 3.2. Cellular Mediators of Perioperative Immune Dysfunction

Postoperative immune dysfunction involves multiple cell populations. NK cells are central to the early elimination of circulating tumor cells, and their suppression has been repeatedly linked to experimental metastasis [5,6,15]. Myeloid-derived suppressor cells (MDSCs) and regulatory T cells (Tregs) can expand in response to tissue injury, inflammatory cytokines, and tumor-derived factors, leading to T-cell dysfunction and impaired antitumor activity [16]. Tumor-associated macrophages may also shift toward phenotypes that promote angiogenesis, matrix remodeling, and immune suppression.

Collectively, these changes reflect a coordinated shift toward inflammation-associated immunosuppression rather than a simple decrease in the number of immune cells. This provides a rationale for agents that normalize the immune balance, restore NK and T helper 1 (Th1) functions, and limit excessive inflammatory tissue injury.

## 4. Molecular and Biological Characteristics of ECLM

ECLM is predominantly composed of carbohydrates (approximately 70%), with smaller proportions of protein, ash, fat, and dietary fiber [17,18,19]; it also contains low-molecular-weight oligosaccharides, including α-glucan-rich fractions, amino acids, minerals, and other small molecules. Although α-glucan-rich fractions have been proposed to contribute to its immunomodulatory activity, the precise bioactive component(s) responsible for the observed biological effects remain unknown. Likewise, the molecular targets and receptor-mediated mechanisms have not been fully elucidated. Thus, ECLM should be regarded as a complex biological mixture rather than a single defined bioactive compound.

Several studies have suggested that ECLM acts as a biological response modifier rather than a direct cytotoxic drug. Human and animal studies have demonstrated broad immunomodulatory activity involving both innate and adaptive immunity [7,8,9,10,11,20]. ECLM is an orally administered immune-modulating product that may activate dendritic cells, NK cells [7], macrophages [21], and T cells [22] through Toll-like receptor (TLR)-related pathways; however, receptor-level mechanisms require further experimental validation. Unlike conventional pharmacological immunomodulators, ECLM is administered orally as a nutritional supplement. One proposed bioactive fraction consists of low-molecular-weight α-1,4-glucans generated during the culture of *Lentinula edodes* mycelia. In comparison with high-molecular-weight β-glucans such as lentinan, these α-glucan-rich fractions may facilitate gastrointestinal interaction with gut-associated lymphoid tissues, thereby contributing to immunomodulatory activity.

In addition to immune regulation, ECLM may also support nutritional status. Clinical studies in patients with pancreatic cancer have demonstrated reductions in chemotherapy-related taste disorders and improvements in nutritional parameters during treatment. These findings suggest that ECLM may support the maintenance of oral intake and host resilience during cancer therapy. Accordingly, ECLM may be considered an immunonutritional intervention rather than an immunomodulatory supplement alone. Although ECLM is manufactured under standardized production conditions, the contribution of individual constituents, potential batch-to-batch variation, and the precise molecular mechanisms underlying its biological effects remain important areas for future investigation.

## 5. Immunomodulatory Mechanisms

### 5.1. NK Cell and Innate Immune Modulation

NK cells are plausible targets for ECLM because they are essential for early tumor surveillance and are highly sensitive to surgical stress. ECLM has been reported to enhance immune parameters in healthy volunteers and animal models [7,9,20]. Studies in mice have also suggested that ECLM supplementation can increase innate immune responsiveness in young and aged animals [9].

The magnitude and reproducibility of NK cell enhancement vary across models and study designs. Therefore, ECLM may preserve or enhance NK cell activity under specific physiological stress conditions rather than claiming universal immune activation. This distinction is particularly important in perioperative oncology, where the therapeutic goal is immune homeostasis rather than overstimulation.

### 5.2. Antigen-Presenting Cells and T-Cell Responses

ECLM has been linked to the modulation of antigen-presenting cells and T-cell functions. In healthy adults, ECLM has been reported to influence the frequency of CD4+ and CD8+ T cells producing interferon-gamma and/or tumor necrosis factor-α [20]. These findings support a potential shift toward Th1-associated immune responses, which may contribute to antitumor immunity.

Preclinical tumor studies have suggested that ECLM can regulate both innate and adaptive immune responses [8]. However, the exact relationships among polysaccharide structure, intestinal immune sensing, antigen-presenting cell activation, and systemic antitumor immunity remain unclear. Future mechanistic studies should include receptor blocking experiments, cell-specific depletion, and single-cell immune profiling.

### 5.3. Cytokine Balance and Immune Normalization

**Figure 1:**
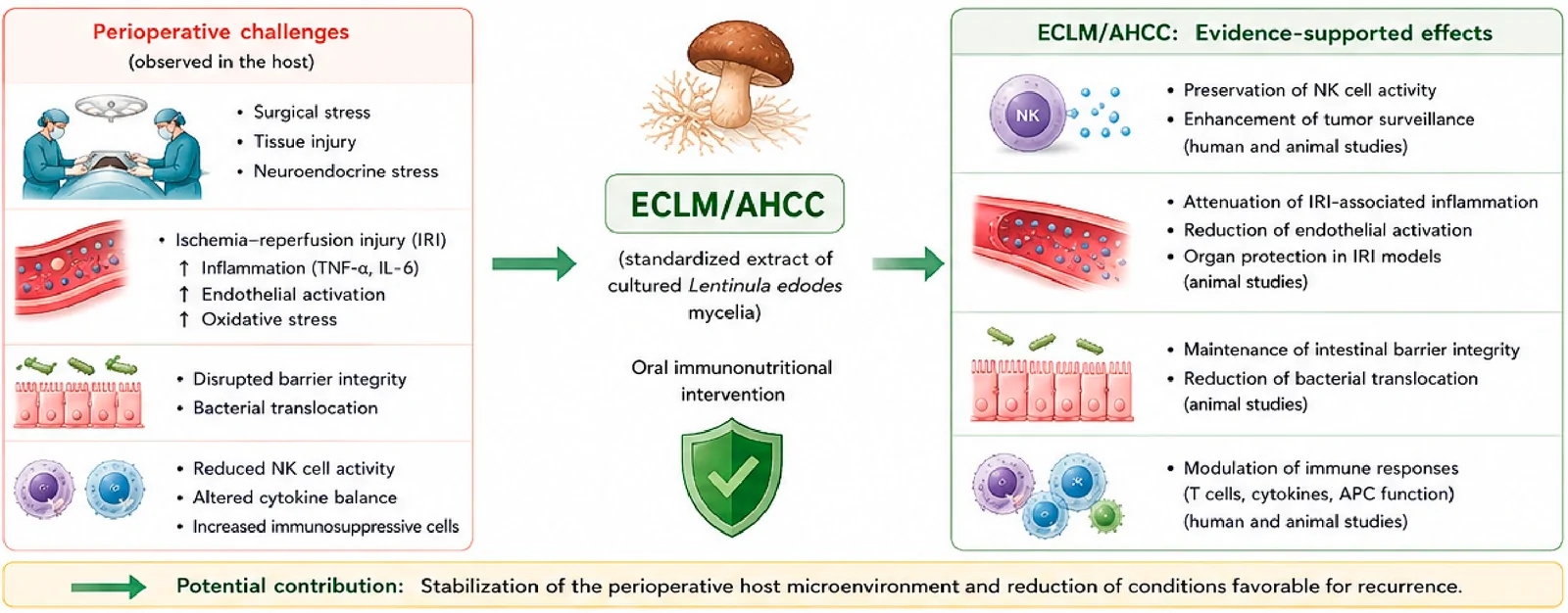
Evidence-supported actions of ECLM relevant to the perioperative period. Perioperative challenges, such as surgical stress, IRI-induced inflammation and endothelial activation, intestinal barrier dysfunction, and immune suppression, have been observed. ECLM has been reported to exert various effects that counteract these processes. These effects may contribute to stabilizing the perioperative microenvironment and reducing metastatic recurrence. Note: This figure summarizes actions supported by published evidence. Causal relationships and clinical benefit require further validation. Abbreviations: APC, antigen-presenting cell; IL, interleukin; TNF, tumor necrosis factor.

An important concept emerging from the literature is that ECLM may function as an immune-modulating agent. In immunosuppressed or stressed conditions, it may support NK and Th1 responses. In inflammatory organ injury models, it appears to reduce excessive inflammatory cytokine signaling [12,13]. This dual behavior distinguishes ECLM from purely pro-inflammatory immune stimulants and may explain its potential perioperative relevance. The evidence-supported effects of ECLM that may be relevant to the perioperative microenvironment are summarized in Figure 1.

## 6. Preclinical Antitumor Evidence

### 6.1. Immune-Mediated Tumor Surveillance

Gao et al. reported that ECLM enhanced tumor surveillance in mice by regulating both innate and adaptive immune responses [8]. These data provide a key mechanistic foundation for the concept that ECLM influences host-mediated antitumor immunity.

The antitumor effect of ECLM in immunocompetent models appears to depend largely on host immune mechanisms. Therefore, the preclinical findings should not be interpreted as evidence of conventional cytotoxicity. Instead, ECLM may enhance immune recognition, effector cell function, or microenvironmental conditions that permit tumor control.

### 6.2. Combination with Chemotherapy

Cao et al. demonstrated that ECLM potentiated the antitumor effects of low-dose 5-fluorouracil (5-FU) in hepatoma 22 tumor-bearing mice by modulating immune function [23]. This finding is conceptually important because chemotherapy can release tumor antigens while simultaneously inducing immunosuppression. ECLM may help preserve immune effector function during cytotoxic therapy.

In pancreatic cancer models, ECLM has been reported to affect gemcitabine-resistant cancer cells, including downregulation of cortactin [24]. These findings suggest that ECLM-related products may influence tumor biology and treatment resistance pathways, although their clinical relevance requires further validation.

Furthermore, administration of ECLM in patients with various cancers receiving chemotherapy has been reported to reduce salivary human herpes virus levels, improve quality of life, and alleviate hematological and hepatic toxicities, suggesting its potential supportive role during chemotherapy [25].

### 6.3. Combination with Immune Checkpoint Blockade

Park et al. reported that oral ECLM promotes the antitumor effect of dual programmed death 1 (PD-1)/cytotoxic T-lymphocyte antigen (CTLA)-4 blockade in MC38 colon cancer-bearing mice. The combination treatment reduced tumor growth and increased granzyme B and Ki-67 expression in tumor-infiltrating CD8+ T cells, and this effect was linked to changes in the gut microbiome [26]. This study suggests that ECLM may act upstream of or in parallel with checkpoint blockade by altering immune competence and microbiome-related responses.

These findings justify the cautious exploration of ECLM in combination with modern immunotherapies. However, translation to humans requires careful trial design, because supplement-immunotherapy interactions can be context-dependent and may be influenced by microbiome composition, cancer type, prior therapy, and host immune status.

## 7. Clinical Evidence in Oncology

### 7.1. Hepatocellular Carcinoma

Studies on hepatocellular carcinoma (HCC) have provided the most clinically relevant evidence for the use of ECLM in oncology. Matsui et al. reported improved prognosis of postoperative HCC patients who received ECLM as a functional food intervention [27]. Cowawintaweewat et al. later reported prognostic improvements in patients with advanced liver cancer who received ECLM [28]. More recently, Kamiyama et al. evaluated ECLM as an adjuvant therapy after curative hepatectomy for advanced HCC in an open-label, single-arm study and reported its feasibility, safety, and encouraging recurrence-related outcomes [29].

These studies are clinically meaningful, but their findings should be interpreted with caution. Several studies were nonrandomized or uncontrolled, and the observed benefits cannot be definitively attributed to ECLM without adequately powered randomized trials. Nevertheless, the pattern of benefit, recurrence prevention, or disease stabilization, rather than rapid tumor shrinkage, is consistent with the concept of host-mediated immune surveillance. No randomized trial has demonstrated improvement in overall survival or recurrence-free survival.

### 7.2. Pancreatic and Gynecologic Malignancies

In a randomized study that examined CD4+ and CD8+ lymphocyte levels, ECLM supplementation was evaluated in patients with epithelial ovarian or peritoneal cancer receiving platinum-based chemotherapy; however, the increase in lymphocyte levels was limited, and no significant effects on bone marrow suppression or quality of life were observed [30]. In pancreatic cancer, a randomized, double-blind, placebo-controlled trial reported that ECLM reduces chemotherapy-related taste disorders and improves nutritional parameters in patients receiving chemotherapy [31]. A double-blind randomized phase II study protocol was also published to evaluate the impact on survival in resectable or borderline resectable pancreatic cancers [32].

These studies support the broader relevance of ECLM in supportive care and immunonutrition in oncology. However, most available data have not yet established improved overall survival across tumor types. Future trials should incorporate immune monitoring and recurrence endpoints rather than relying solely on quality-of-life or nutritional outcomes.

## 8. ECLM Protects Against IRI and Perioperative Tumor-Promoting Microenvironments

IRI commonly occurs during liver resection, vascular clamping, transplantation, pancreatic surgery, and other major abdominal procedures. During ischemia, adenosine triphosphate (ATP) depletion and mitochondrial dysfunction occur, whereas reperfusion triggers the production of reactive oxygen species, complement activation, neutrophil infiltration, cytokine release, and endothelial activation. From an oncological perspective, IRI is not merely an organ injury phenomenon but may also contribute to the establishment of a pro-metastatic microenvironment through endothelial adhesion molecule expression, extracellular matrix remodeling, inflammatory cytokine production, and recruitment of immunosuppressive myeloid cells. These changes may facilitate circulating tumor cell adhesion, survival, and metastatic outgrowth.

### 8.1. Intestinal IRI Model

Ueyama et al. investigated ECLM in a rat model of small intestinal IRI and demonstrated its protective effects against reperfusion-associated injuries [12]. Although this study was not designed as a cancer recurrence model, it provided important mechanistic evidence supporting the organ-protective and immunonutritional properties of ECLM. Preservation of intestinal barrier integrity may reduce bacterial translocation, maintain nutrient absorption, and attenuate systemic inflammation, thereby limiting postoperative immune dysfunction and metabolic stress. These findings suggest that ECLM may contribute to the maintenance of gastrointestinal function and nutritional homeostasis during the perioperative period, thereby supporting its potential role as an organ-protective immunonutritional intervention in surgical oncology.

### 8.2. Hepatic IRI and Partial Hepatectomy Model

Nakatake et al. reported that ECLM protects against liver injury induced by IRI and partial hepatectomy [13]. This model is highly relevant to surgery for HCC and colorectal liver metastases, in which hepatic inflow occlusion and parenchymal transection frequently induce inflammatory liver injury. The observed hepatoprotective effects support a dual-action hypothesis wherein ECLM preserves host immune competence while simultaneously reducing inflammation-driven organ damage.

Collectively, these findings suggest that attenuation of IRI may represent an additional mechanism by which ECLM influences postoperative oncological outcomes. By reducing inflammatory organ injury, preserving intestinal barrier function, and maintaining a less permissive perioperative microenvironment, ECLM may contribute to the prevention of recurrence beyond its direct immunomodulatory effects.

## 9. Bench-to-Bedside Development: PDX Models and Prevention

### 9.1. Patient-Derived Xenograft Models

Yoshii et al. used Super severe combined immunodeficiency (SCID) mice bearing patient-derived xenografts to investigate tumor growth inhibition by ECLM [33]. The authors reported significant suppression of renal cancer PDX growth during a part of the observation period and growth-inhibitory tendencies in breast cancer PDX models [33]. Since Super SCID mice lack functional adaptive immunity and have profoundly impaired immune responses, these findings suggest that ECLM may exert effects beyond conventional T-cell-mediated antitumor immunity.

However, the PDX data should be interpreted cautiously. Super SCID models do not completely eliminate innate immune or stromal influences, and the underlying mechanisms of tumor suppression were not directly investigated. Thus, the antitumor effects of ECLM may not be entirely dependent on adaptive immunity and may involve additional host- or tumor-related mechanisms. Nevertheless, the precise pathways involved remain unclear and require further mechanistic investigations.

### 9.2. Cancer-Prevention Models

Adachi et al. reported that continuous ingestion of ECLM suppressed spontaneous carcinogenesis in C3H/HeJ mice [34]. Long-term ingestion delayed the first death, improved survival rates, increased the proportion of mice without organ abnormalities, and reduced the incidence of cancer-bearing mice, particularly breast and liver cancers [34].

**Figure 2:**
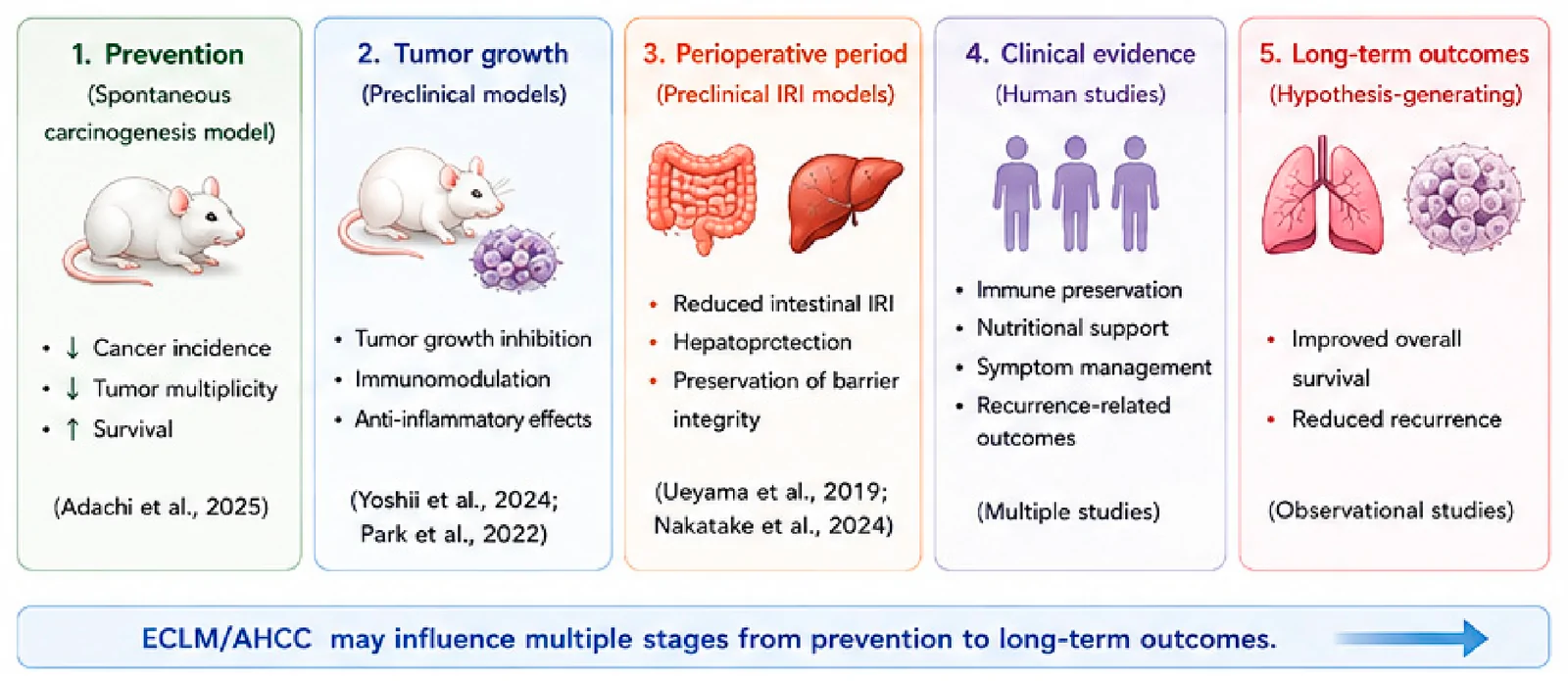
Summary of current evidence across the cancer continuum. Cancer-control continuum illustrating the proposed multistage actions of ECLM across the cancer trajectory. Evidence from spontaneous carcinogenesis models to human studies suggests that ECLM may influence multiple stages of cancer, from prevention to long-term outcomes. The figure presents a hypothesis-generating conceptual model that integrates observations from independent experimental and clinical studies and does not imply a validated biological pathway or established causal relationships [12,13,26,33,34]. Note: Evidence levels vary by stage. Human data consist mainly of observational or small intervention studies. Causal relationships cannot be inferred.

These data broaden the biological scope of ECLM from perioperative recurrence prevention to earlier stages of cancer development. They suggest that ECLM may influence the continuum from chronic inflammation and tumor initiation to progression and postoperative recurrence. This multistage concept is highly relevant to nutrition because it links functional food biology with cancer prevention and supportive oncology. The proposed multistage cancer-control continuum of the ECLM is shown in Figure 2.

### 9.3. Integrated Translational Hypothesis: From Immunonutrition to Perioperative Microenvironment Stabilization

Combined evidence from PDX models, spontaneous carcinogenesis studies, IRI models, and clinical hepatocellular carcinoma cohorts supports a multilevel model of ECLM action. At the host level, ECLM may preserve NK cell- and Th1-mediated tumor surveillance, thereby maintaining immune competence during periods of physiological stress. At the organ level, ECLM attenuates IRI-associated inflammation and tissue injury, preserving intestinal barrier integrity and reducing the formation of a pro-metastatic perioperative microenvironment. At the tumor-intrinsic level, emerging PDX studies have suggested modulation of cellular stress responses, metabolic adaptation, and tumor growth dynamics.

Recent advances in patient-derived cancer models have provided unique opportunities to bridge the gap between nutritional science and precision oncology. Notably, the antitumor activity observed in PDX models established in severely immunodeficient mice suggests that the biological effects of ECLM are not entirely dependent on adaptive immune activation. Because these models lack functional T-cell responses, the observed tumor growth inhibition may reflect the direct or indirect regulation of tumor cell biology, oxidative stress pathways, metabolic homeostasis, or tumor–stromal interactions. However, these mechanisms are poorly understood and warrant further investigation.

**Figure 3:**
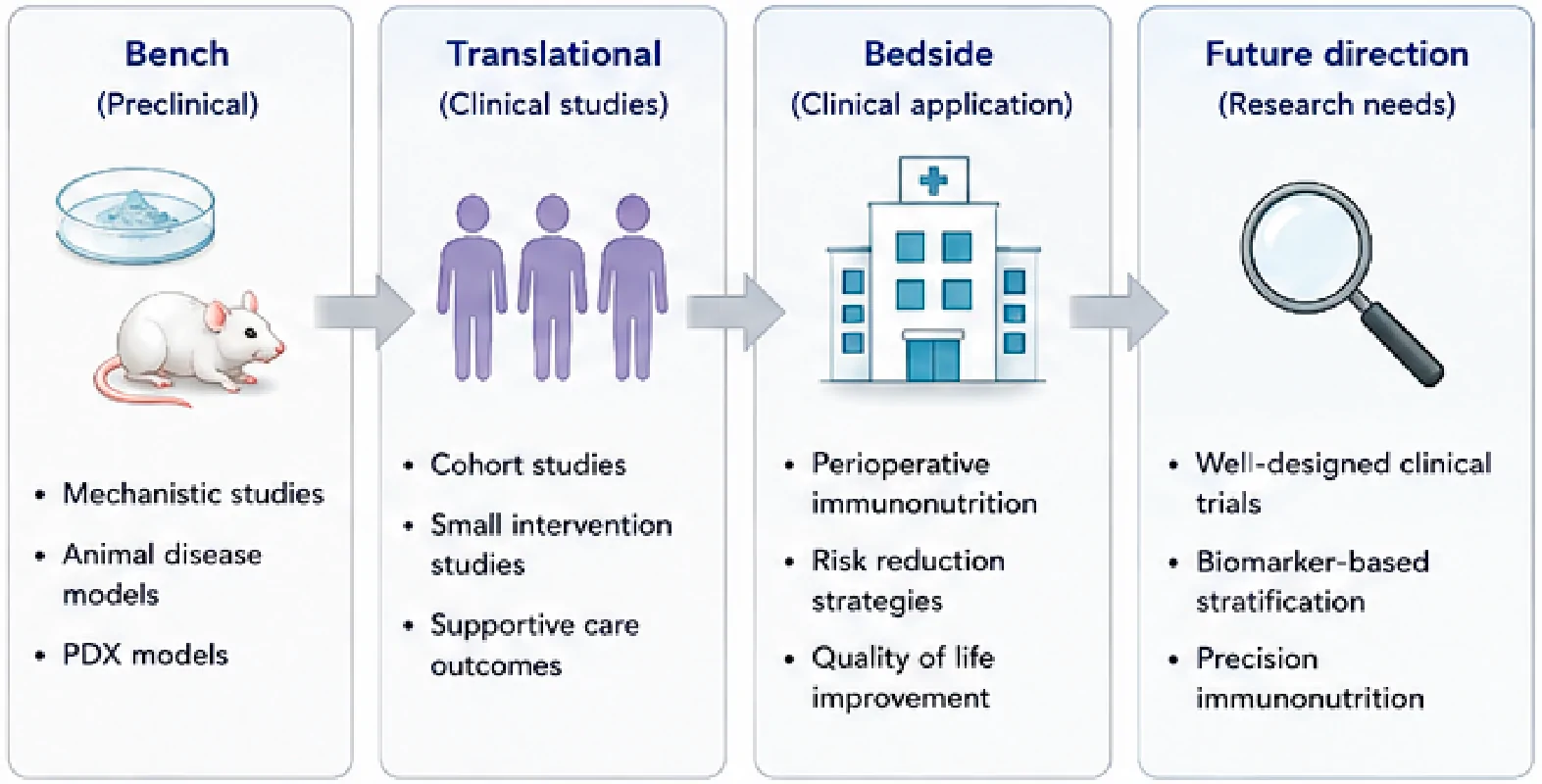
From bench to bedside: Current evidence and future direction. This framework illustrates the translational progression of ECLM from preclinical to clinical studies, and the underlying evidence supports future hypothesis-driven perioperative clinical trials that integrate immune preservation, organ protection, and oncological outcomes. Through multiple stages, this framework ultimately serves as a guide for future research and development as well as clinical application. Note: Many links in this continuum are hypothetical and require further validation. This figure illustrates a conceptual framework rather than an established pathway.

Collectively, these independent lines of evidence suggest the possibility that ECLM may influence multiple aspects of host–tumor interactions. However, these observations originate from heterogeneous experimental systems and should not be interpreted as evidence of a single unified biological mechanism. Rather, they provide a hypothesis-generating framework that warrants further mechanistic investigation. Future translational studies that incorporate PDX validation, perioperative immune monitoring, circulating tumor cell assessment, and recurrence-free survival endpoints may establish a new paradigm linking functional foods, immunonutrition, and precision cancer therapies. The proposed bench-to-bedside translational pathway is illustrated in Figure 3.

Taken together, these observations suggest the idea that ECLM can serve as a modulator of the perioperative microenvironment through the aforementioned immune-enhancing mechanisms, contributing to attenuation of IRI and potential regulation of tumor biology. This multistage mode of action may explain the emerging clinical benefits observed for cancer prevention, perioperative management, and long-term oncological outcomes (Table 1).

## 10. Perioperative Oncologic Implications

The central hypothesis of this review was that surgery produces two co-operating pro-metastatic processes: immune suppression and inflammatory organ injury. ECLM may interrupt both processes by preserving immune surveillance and attenuating IRI-associated inflammation.

A practical perioperative research protocol could include preoperative administration for one to two weeks, continuation after surgery, and maintenance during the early recurrence-prone period. However, dosing and duration should be tested prospectively, and current evidence is insufficient to recommend routine perioperative use as standard care.

## 11. Safety, Drug Interactions, and Cautions

ECLM has generally shown favorable tolerability in clinical studies, including in supportive care settings for HCC and pancreatic cancer [28,29,31]. Pharmacokinetic evaluation suggests a low potential for interactions with common chemotherapeutic agents, although supplement–drug interactions remain an important consideration in oncology [35].

Because ECLM may modulate immune function, caution is warranted in patients receiving immunosuppressive therapy, transplant recipients, and patients with active autoimmune diseases. Perioperative studies should prospectively monitor adverse events, liver and renal function, coagulation, infection, and wound healing.

## 12. Limitations of Current Evidence

This study had several limitations. First, many clinical studies were small, single-center, non-randomized, or uncontrolled. Second, the mechanistic data are heterogeneous and often derived from different ECLM preparations, doses, and models. Third, the immune endpoints were inconsistently measured, making cross-study comparisons difficult. Fourth, PDX findings are promising but do not yet define the exact tumor-intrinsic pathways responsible for growth suppression. Potential publication and commercial bias should also be considered. Much of the currently available literature on ECLM originates from a limited number of research groups, and several clinical studies have involved commercial sponsorship or industry collaboration. Although this does not invalidate the reported findings, these factors should be transparently acknowledged when interpreting the current evidence. In addition, the precise bioactive components responsible for the observed biological effects remain incompletely characterized, and the relative contributions of α-glucans, amino acids, and other constituents require further investigation. Independent replication by external investigators and large, well-designed randomized controlled trials are required to establish the generalizability and clinical relevance of the observed effects.

## 13. Future Directions

Future studies should prioritize mechanism-driven translational designs. For perioperative gastrointestinal cancers, randomized trials should evaluate recurrence-free survival together with serial NK cell activity, CD8+ T-cell function, MDSC frequency, and the levels of inflammatory cytokines, IRI markers, and circulating tumor cells. The most compelling future strategy would be the development of a bench-to-bedside platform that integrates PDX validation, perioperative biomarker monitoring, and prospective clinical testing. Such a framework would allow ECLM to be evaluated as a biologically rational immunonutritional intervention, rather than as an empirical supplement. Although findings from spontaneous carcinogenesis models, PDX studies, and perioperative experimental models are collectively informative, they represent distinct experimental settings with different biological contexts. Therefore, the proposed cancer continuum should be regarded as a conceptual framework rather than an experimentally validated sequence of events.

## 14. Conclusions

ECLM is a standardized mushroom-derived extract with immunomodulatory, organ-protective, and potentially tumor-regulatory properties. The existing evidence suggests that ECLM may preserve NK- and Th1-mediated immune surveillance, attenuate intestinal and hepatic IRI, and potentially support selected anticancer therapies. Its most compelling translational application is in perioperative oncology, where immune suppression and IRI contribute to postoperative recurrence.

Emerging evidence from PDXs and spontaneous carcinogenesis models provides preliminary support for the hypothesis that ECLM may influence multiple stages of cancer biology, including tumor initiation, progression, and recurrence. Accordingly, ECLM may be viewed not simply as an immunostimulatory supplement but also as a modulator of perioperative homeostasis.

Although the current evidence is insufficient for routine clinical use, these findings support further development of ECLM administration as a novel immunonutritional strategy spanning cancer prevention, perioperative care, and recurrence prevention. Future randomized trials incorporating immunity, organ injury, and oncological endpoints are warranted to validate the role of ECLM in the emerging field of precision immunonutrition.

## Figures and Tables

**Table 1:** Representative evidence supporting ECLM in oncology and perioperative biology.

Evidence Layer	Study Design	Model/Setting	Sample Size	Key Findings	Interpretation	Ref
Immune modulation	Double-blind, placebo-controlled trial	Healthy volunteers	21 (ECLM 11, control 10)	Changes in NK cell activity and T-cell cytokine responses	Supports the biological response modifier concept	[7]
Tumor surveillance	Controlled animal study	Murine tumor models	5 mice/group	Enhanced tumor surveillance via innate and adaptive immunity	Host-mediated antitumor mechanism	[8]
Immune modulation	Controlled animal study	Murine influenza model	8 mice/group	Improved innate immune response to acute influenza infection	Supports the biological response modifier concept	[9]
Immune modulation	Controlled animal study	Young murine influenza model	20 mice/group	Increased innate immune response during primary influenza infection	Supports the biological response modifier concept	[10]
Surgical wound infection	Controlled animal study	Murine surgical wound infection model	20 mice/group	Enhanced resistance to infection	Supports the biological response modifier concept	[11]
Immune modulation	Clinical intervention study	Healthy volunteers	30 (ECLM 15, control 15)	Increased frequency of IFN-γ/TNF-α-producing CD4^+^ and CD8^+^ T cells	Supports the biological response modifier concept	[20]
Chemotherapy combination	Controlled animal study	Murine tumor models	10 mice/group	Potentiation of the antitumor effects of low-dose 5-FU	Possible immune support during cytotoxic therapy	[23]
Chemotherapy supportive care	Prospective self-controlled clinical intervention study	Advanced cancer receiving chemotherapy	24 patients	Reduced salivary HHV-6 DNA levels; improved QOL; attenuated hepatotoxicity	Possible immune support during cytotoxic therapy	[25]
Checkpoint blockade	Controlled animal study	Murine tumor models	5–6 mice/group	Enhanced effect of dual PD-1/CTLA-4 blockade and altered microbiome	Potential immunotherapy combination rationale	[26]
HCC clinical studies	Prospective cohort	HCC after curative hepatectomy	269 patients	Improved prognosis or encouraging recurrence-related outcomes	Hypothesis-generating clinical support	[27]
HCC clinical studies	Prospective cohort	Advanced HCC	44 (ECLM 34; control 10)	Prolonged survival; improved QOL	Hypothesis-generating clinical support	[28]
HCC clinical studies	Single-arm, open-label study	HCC after curative hepatectomy	29 patients	Feasibility; exploratory recurrence outcomes	Hypothesis-generating clinical support	[29]
Pancreatic cancer clinical studies	Randomized, double-blind, placebo-controlled trial	Pancreatic cancer receiving chemotherapy	98 patients	Reduced taste disorders; improved nutrition	Hypothesis-generating clinical support	[31]
Ovarian/peritoneal cancer clinical studies	Randomized, double-blind, placebo-controlled trial	Ovarian/peritoneal cancer receiving platinum-based chemotherapy	32 randomized (28 analyzed)	Not increased CD4^+^ or CD8^+^ T-cells; reduced nausea/vomiting	Hypothesis-generating clinical support	[30]
Intestinal IRI	Controlled animal study	Rat small intestine IRI	6 rats/group	Reduced reperfusion-associated intestinal injury	Perioperative organ-protection rationale	[12]
Hepatic IRI	Controlled animal study	Rat partial hepatectomy + IRI	Main study: 29 rats; survival study: 20 rats	Reduced liver injury after IRI and hepatectomy	Direct relevance to hepatobiliary surgery	[13]
PDX	PDX study in severely immunodeficient mice	Super SCID mouse PDX	Renal PDX: 5 mice/group; Breast PDX: 5–10 mice/group	Suppressed renal cancer PDX growth; trends in breast cancer PDX	Possible tumor-intrinsic or partially immune-independent effects	[33]
Prevention	Long-term controlled animal study	Spontaneous carcinogenesis model	72–109 mice (30–41 females, 27–68 males)	Reduced cancer-bearing mice and improved survival	Supports multistage cancer-control hypothesis	[34]

Abbreviations: 5-FU, 5-fluorouracil; CTLA, cytotoxic T-lymphocyte antigen; HCC, hepatocellular carcinoma; HHV, human herpes viruses; IRI, ischemia–reperfusion injury; NK, natural killer; PD-1, programmed death 1; PDX, patient-derived xenograft; QOL, quality of life; SCID, severe combined immunodeficiency.

## Data Availability

No new data were created or analyzed in this study. Data sharing is not applicable to this article.

## Abbreviations

The following abbreviations are used in this manuscript:

CTC	Circulating tumor cell
ECLM	A standardized extract of cultured *Lentinula edodes* mycelia
HCC	Hepatocellular carcinoma
IRI	Ischemia–reperfusion injury
NK	Natural killer
PDX	Patient-derived xenograft
SCID	Severe combined immunodeficiency

## References

[1] Hanahan D., Weinberg R.A. (2011). Hallmarks of cancer: The next generation. Cell.

[2] Horowitz M., Neeman E., Sharon E., Ben-Eliyahu S. (2015). Exploiting the critical perioperative period to improve long-term cancer outcomes. Nat. Rev. Clin. Oncol..

[3] Tohme S., Simmons R.L., Tsung A. (2017). Surgery for Cancer: A Trigger for Metastases. Cancer Res..

[4] Coffey J.C., Wang J.H., Smith M.J., Bouchier-Hayes D., Cotter T.G., Redmond H.P. (2003). Excisional surgery for cancer cure: Therapy at a cost. Lancet Oncol..

[5] Ben-Eliyahu S., Page G.G., Yirmiya R., Shakhar G. (1999). Evidence that stress and surgical interventions promote tumor development by suppressing natural killer cell activity. Int. J. Cancer.

[6] Shakhar G., Ben-Eliyahu S. (2003). Potential prophylactic measures against postoperative immunosuppression: Could they reduce recurrence rates in oncological patients?. Ann. Surg. Oncol..

[7] Terakawa N., Matsui Y., Satoi S., Yanagimoto H., Takahashi K., Yamamoto T., Yamao J., Takai S., Kwon A.H., Kamiyama Y. (2008). Immunological effect of active hexose correlated compound (AHCC) in healthy volunteers: A double-blind, placebo-controlled trial. Nutr. Cancer.

[8] Gao Y., Zhang D., Sun B., Fujii H., Kosuna K., Yin Z. (2006). Active hexose correlated compound enhances tumor surveillance through regulating both innate and adaptive immune responses. Cancer Immunol. Immunother..

[9] Nogusa S., Gerbino J., Ritz B.W. (2009). Low-dose supplementation with active hexose correlated compound improves the immune response to acute influenza infection in C57BL/6 mice. Nutr. Res..

[10] Ritz B.W., Nogusa S., Ackerman E.A., Gardner E.M. (2006). Supplementation with active hexose correlated compound increases the innate immune response of young mice to primary influenza infection. J. Nutr..

[11] Aviles H., O’Donnell P., Sun B., Sonnenfeld G. (2006). Active hexose correlated compound (AHCC) enhances resistance to infection in a mouse model of surgical wound infection. Surg. Infect..

[12] Ueyama Y., Tokuhara K., Miki H., Nakatake R., Sakaguchi T., Nishizawa M., Kaibori M., Okumura T. (2019). Active Hexose Correlated Compound Has Protective Effects in Ischemia-Reperfusion Injury of the Rat Small Intestine. J. Surg. Res..

[13] Nakatake R., Okuyama T., Ishizaki M., Yanagida H., Kitade H., Yoshizawa K., Nishizawa M., Sekimoto M. (2024). Hepatoprotection of a Standardized Extract of Cultured *Lentinula edodes* Mycelia against Liver Injury Induced by Ischemia-Reperfusion and Partial Hepatectomy. Nutrients.

[14] Hiller J.G., Perry N.J., Poulogiannis G., Riedel B., Sloan E.K. (2018). Perioperative events influence cancer recurrence risk after surgery. Nat. Rev. Clin. Oncol..

[15] Bezu L., Akcal Oksuz D., Bell M., Buggy D., Diaz-Cambronero O., Enlund M., Forget P., Gupta A., Hollmann M.W., Ionescu D. (2024). Perioperative Immunosuppressive Factors during Cancer Surgery: An Updated Review. Cancers.

[16] Talmadge J.E., Gabrilovich D.I. (2013). History of myeloid-derived suppressor cells. Nat. Rev. Cancer.

[17] Sato K., Kishimoto M. (2016). Composition. Clinician’s Guide to AHCC: Evidence-Based Nutritional Immunotherapy.

[18] Miura T., Kitadate K., Nishioka H., Wakame K. (2010). Basic and Clinical Studies on Active Hexose Correlated Compound.

[19] Fujii H., Nishioka N., Simon R.R., Kaur R., Lynch B., Roberts A. (2011). Genotoxicity and subchronic toxicity evaluation of Active Hexose Correlated Compound (AHCC). Regul. Toxicol. Pharmacol..

[20] Yin Z., Fujii H., Walshe T. (2010). Effects of active hexose correlated compound on frequency of CD4^+^ and CD8^+^ T cells producing interferon-gamma and/or tumor necrosis factor-alpha in healthy adults. Hum. Immunol..

[21] Daddaoua A., Martinez-Plata E., Ortega-Gonzalez M., Ocon B., Aranda C.J., Zarzuelo A., Suarez M.D., de Medina F.S., Martinez-Augustin O. (2013). The nutritional supplement Active Hexose Correlated Compound (AHCC) has direct immunomodulatory actions on intestinal epithelial cells and macrophages involving TLR/MyD88 and NF-kappaB/MAPK activation. Food Chem..

[22] Olamigoke L., Mansoor E., Mann V., Ellis I., Okoro E., Wakame K., Fuji H., Kulkarni A., Francoise Doursout M., Sundaresan A. (2015). AHCC Activation and Selection of Human Lymphocytes via Genotypic and Phenotypic Changes to an Adherent Cell Type: A Possible Novel Mechanism of T Cell Activation. Evid.-Based Complement. Altern. Med..

[23] Cao Z., Chen X., Lan L., Zhang Z., Du J., Liao L. (2015). Active hexose correlated compound potentiates the antitumor effects of low-dose 5-fluorouracil through modulation of immune function in hepatoma 22 tumor-bearing mice. Nutr. Res. Pract..

[24] Islam S., Kitagawa T., Baron B., Kuhara K., Nagayasu H., Kobayashi M., Chiba I., Kuramitsu Y. (2021). A standardized extract of cultured *Lentinula edodes* mycelia downregulates cortactin in gemcitabine-resistant pancreatic cancer cells. Oncol. Lett..

[25] Ito T., Urushima H., Sakaue M., Yukawa S., Honda H., Hirai K., Igura T., Hayashi N., Maeda K., Kitagawa T. (2014). Reduction of adverse effects by a mushroom product, active hexose correlated compound (AHCC) in patients with advanced cancer during chemotherapy—The significance of the levels of HHV-6 DNA in saliva as a surrogate biomarker during chemotherapy. Nutr. Cancer.

[26] Park H.J., Boo S., Park I., Shin M.S., Takahashi T., Takanari J., Homma K., Kang I. (2022). AHCC((R)), a Standardized Extract of Cultured *Lentinula edodes* Mycelia, Promotes the Anti-Tumor Effect of Dual Immune Checkpoint Blockade Effect in Murine Colon Cancer. Front. Immunol..

[27] Matsui Y., Uhara J., Satoi S., Kaibori M., Yamada H., Kitade H., Imamura A., Takai S., Kawaguchi Y., Kwon A.H. (2002). Improved prognosis of postoperative hepatocellular carcinoma patients when treated with functional foods: A prospective cohort study. J. Hepatol..

[28] Cowawintaweewat S., Manoromana S., Sriplung H., Khuhaprema T., Tongtawe P., Tapchaisri P., Chaicumpa W. (2006). Prognostic improvement of patients with advanced liver cancer after active hexose correlated compound (AHCC) treatment. Asian Pac. J. Allergy Immunol..

[29] Kamiyama T., Orimo T., Wakayama K., Kakisaka T., Shimada S., Nagatsu A., Asahi Y., Aiyama T., Kamachi H., Taketomi A. (2022). Preventing Recurrence of Hepatocellular Carcinoma After Curative Hepatectomy with Active Hexose-correlated Compound Derived from *Lentinula edodes* Mycelia. Integr. Cancer Ther..

[30] Suknikhom W., Lertkhachonsuk R., Manchana T. (2017). The Effects of Active Hexose Correlated Compound (AHCC) on Levels of CD4^+^ and CD8^+^ in Patients with Epithelial Ovarian Cancer or Peritoneal Cancer Receiving Platinum Based Chemotherapy. Asian Pac. J. Cancer Prev..

[31] Yanagimoto H., Hirooka S., Yamamoto T., Yamaki S., Sekimoto M. (2023). Efficacy of *Lentinula edodes* Mycelia Extract on Chemotherapy-Related Tasted Disorders in Pancreatic Cancer Patients. Nutr. Cancer.

[32] Hashimoto D., Satoi S., Ishikawa H., Kodera Y., Kamei K., Hirano S., Fujii T., Uemura K., Tsuchida A., Yamada S. (2022). Efficacy of active hexose correlated compound on survival of patients with resectable/borderline resectable pancreatic cancer: A study protocol for a double-blind randomized phase II study. Trials.

[33] Yoshii R., Adachi S., Ryo H., Hatanaka E., Yasuda K., Kaji M., Enomoto K., Takanari J., Yoshidome K., Nomura T. (2024). The Tumor Growth Inhibitory Effect of a Standardized Extract of Cultured *Lentinula edodes* Mycelia Using Patient Derived Xenograft Model. Biol. Pharm. Bull..

[34] Adachi S., Tougan T., Ryo H., Takanari J., Wakame K., Aoshi T., Nomura T. (2025). Continuous Ingestion of a Standardized Extract of Cultured *Lentinula edodes* Mycelia Suppresses Spontaneous Carcinogenesis in a Murine Model. Biol. Pharm. Bull..

[35] Mach C.M., Fugii H., Wakame K., Smith J. (2008). Evaluation of active hexose correlated compound hepatic metabolism and potential for drug interactions with chemotherapy agents. J. Soc. Integr. Oncol..

